# Plant Productivity and Leaf Starch During Grain Fill Is Linked to QTL Containing Flowering Locus T1 (*FT1*) in Wheat (*Triticum aestivum* L.)

**DOI:** 10.3390/plants14040512

**Published:** 2025-02-07

**Authors:** Alanna J. Oiestad, Nancy K. Blake, Brandon J. Tillett, Sergei T. O’Sullivan, Jason P. Cook, Michael J. Giroux

**Affiliations:** Department of Plant Sciences and Plant Pathology, Montana State University, 119 Plant Bioscience Building, Bozeman, MT 59717-3150, USA; alanna.schlosser@montana.edu (A.J.O.); nkblake@gmail.com (N.K.B.); brandon.tillett@montana.edu (B.J.T.); sergeiosullivan@montana.edu (S.T.O.); jason.cook3@montana.edu (J.P.C.)

**Keywords:** anthesis, *FLOWERING LOCUS T* (*FT-1*), grain fill, metabolism, productivity, transitory starch, *Vrn-3D*, wheat, yield

## Abstract

Shifts in the environment due to climate change necessitate breeding efforts aimed at adapting wheat to longer, warmer growing seasons. In this study, 21 modern wheat (*Triticum aestivum* L.) cultivars and 29 landraces were screened for flag leaf starch levels, with the goal of identifying a genetic marker for targeted breeding. The landrace PI 61693 was identified as having exceptionally high flag leaf starch values. Yield trials were carried out in a Berkut × PI 61693 recombinant inbred line (RIL) population and a negative correlation was observed between leaf starch, flowering time, and yield. Genetic mapping identified a Quantitative Trait Loci (QTL) explaining 22–34% variation for leaf starch, flowering time, biomass, and seed yield. The starch synthase *TraesCS7D02G117800* (*wSsI-1*) is located in this region, which possibly accounts for leaf starch variation in this population; also within this QTL is *TraesCS7D02G111600* (*FT-D1*). Sequencing of *FT-D1* identified a single base pair deletion in the 3rd exon of the Berkut allele. This indel has recently been shown to significantly impact flowering time and productivity, and likely led to significant variation in flowering date and yield in this population. Here, we illustrate how allelic selection of *FT-D1* within breeding programs may aid in adapting wheat to changing environments.

## 1. Introduction

Global temperatures are on the rise and previously predictable patterns in temperature and precipitation become more confounded every year [1]. The shift in climate brings challenges to agriculture, a practice that relies on a reasonable amount of predictability. It is estimated from wheat (*Triticum aestivum* L.) cropping models that for every 1 °C increase in temperature globally, the average production of wheat worldwide will decrease by 6% [2]. Cereal breeding efforts focus primarily on increasing grain yield or grain quality traits. In recent years, there has also been an emphasis on focusing on traits that lead to sustainable improvements in the face of a changing climate. It is increasingly difficult to select single genes that lead to crop improvement. A more sustainable approach may be to focus on the interaction of source and sink strength [3].

In cereals, sink strength in the endosperm is largely driven by starch biosynthesis, where starch constitutes 60% or more of seed weight. Starch biosynthesis in the form of source strength is also limiting to plant growth. Leaf starch is accumulated in source tissues during the light period when excess photosynthate is stored as transitory starch. It is then remobilized to sink tissues during the dark period for growth, development, or storage [4]. The starch biosynthesis pathway is controlled primarily by three enzymes: ADP-glucose pyrophosphorylase (AGPase), starch synthase (SS), and starch-branching enzyme (SBE) [5]; each of these enzymes exist as multiple isozymes [6]. The rate-limiting reaction in both source and sink tissues is controlled by AGPase. In this reaction, glucose-1-phosphate and ATP are converted to ADP-glucose and inorganic phosphate [7]. ADP-glucose is then polymerized by SS to produce amylose, a relatively unbranched glucose polymer, and SBEs are responsible for the production of the highly branched glucose polymer amylopectin.

Given that AGPase is the rate limiting factor in the biosynthesis of starch, it has been the focus of most research regarding the interaction of starch and cereal yield. This includes studies that have demonstrated a reduction in AGPase activity at high temperatures in cereals [8]. This supports the hypothesis that selecting for elevated starch levels in cereal leaves may lead to grain yield gains, or at least protect against yield losses as temperatures increase. Wheat genotypes with improved leaf starch biosynthesis may play an important role in maintaining high grain yields in a warming climate.

Many studies have had success in increasing cereal seed size or yield transgenically via overexpression of AGPase under the control of tissue-specific promoters. Increased seed size has been reported in rice (*Oryza sativa* L.) [9] and maize (*Zea mays* L.) [10,11] with endosperm-specific overexpression of AGPase. A similar approach, using the large endosperm-specific *Sh2* promoter, led to increased seed set, rather than seed size, in wheat (*Triticum aestivum* L.) [12,13], rice [14], and maize [15]. Increased seed number was shown to be the result of *Sh2* promoter-conditioned expression in maternal tissues [15,16]. Increased seed set was accompanied by increased plant biomass in rice and wheat [12,13,14], though biomass was not recorded for maize.

Observations in which both seed yield and biomass were increased simultaneously indicate that source strength, rather than sink strength, might be just as or more limiting to plant productivity. This is supported by the observation that photosynthetic rates were enhanced shortly before and after anthesis in wheat plants with *Sh2*-conditioned over-expression of AGPase in seeds [17]. To examine this hypothesis, several studies examined the role of leaf AGPase in cereal leaf tissue. In maize plants with the transposon-derived mutant *agps-m1*, having no leaf starch had a 30% yield reduction compared to wild-type (WT) sister plants, demonstrating that lack of leaf starch is devastating to plant growth [18]. Meanwhile, transgenic studies demonstrated that overexpression of AGPase in leaves often leads to enhanced plant growth and yield. In lettuce, expression of the modified potato (*Solanum tuberosum* L.) AGPase large subunit, *upreg-1*, in leaves led to significantly increased fresh weight [19]. Expression of this transgene in rice leaves also led to enhanced plant productivity [20]. Another rice study observed a 9% seed yield increase with expression of the transgene *Sh2r6hs* in leaves, accompanied by a 30% increase in mature plant biomass [21]. An additional study in rice with simultaneous upregulation of AGPase in both leaf (*Sh2r6hs* and *Bt2* under *rbcS* promoter) and seed tissue (*Sh2r6hs* under *Sh2* promoter) observed an additive increase in seed yield and biomass compared to overexpression in leaf or seed tissue alone [22].

Most research regarding plant yield linked to starch biosynthesis has focused on AGPase by either modifying AGPase expression levels, manipulating allosteric properties, or altering substrate/product supply [5]. Other starch biosynthesis enzymes have received less attention. In cereals, studies regarding SSs and SBEs have focused on the complex interactions observed within protein complexes, and how they contribute to grain quality. There has been less focus on targeting SS and SBE to increase growth and yield, especially in cereal leaves. However, constitutive overexpression of *SSIV* in leaves of *Arabidopsis thaliana* led to both increased starch and biomass [23], and constitutive upregulation of SBEs led to increased leaf starch and seed yield [24]. Although not immediately transferrable to cereal crops, these observations lend support to the hypothesis that transitory starch biosynthesis may boost plant productivity through multiple pathways.

In further support of this hypothesis, recent research into the transcriptional regulation of starch metabolism has shown that transcription factors from many major families directly regulate cereal starch biosynthesis [25,26,27]. These include the bZIP, AP2/ERF, NAC, CUC2, MYB, GRAS, and DOF transcription factor families, where each was shown to influence starch metabolism in rice, wheat, maize, or barley. Several studies have shown altered levels of starch when individual transcription factors were overexpressed [28,29,30,31,32]; however, due to the complex nature of transcriptional regulation, responses often varied depending on the environment. Furthermore, responses vary between species, even within the cereals [27].

The goal of this study was to identify natural sources of flag leaf starch in spring wheat and correlate leaf starch with plant productivity to identify markers for targeted breeding. It is expected that elevated leaf starch levels indicate a more efficient starch biosynthesis pathway that can be exploited to maintain yields under increasing temperatures. Landraces are excellent resources both in terms of introducing genetic diversity to current cultivars as well as for examining the role of individual genes upon specific traits. Here, both modern cultivars and landraces were screened to identify genotypes with high levels of leaf starch at grain fill. Focus was placed on grain fill since this is the ideal stage to optimize the source–sink strength relationship, and variation in leaf starch at grain fill has been shown to be particularly important to yield [22]. After identifying a landrace with exceptionally high levels of leaf starch, a recombinant inbred line (RIL) population was used to identify a marker associated with 22–34% of the variation for leaf starch, flowering time, biomass, and seed yield. Within this QTL, the starch synthase gene *TraesCS7D02G117800* (*wSsI-1*) was identified as a possible candidate gene for leaf starch differences. *TraesCS7D2G111600* (*FT-D1*), a known flowering time gene, is also located within this QTL. Furthermore, an *FT-D1* indel was identified that segregated with leaf starch, flowering date, and wheat productivity. These findings add to the body of research indicating that FT1 allelic variation may prove useful in selecting specific flowering dates and yields targeted at specific growing regions and environments. This will be an especially important consideration when selecting sustainable yield traits in a changing climate.

## 2. Results

### 2.1. Survey of Leaf and Seed Starch at Grain Fill Identifies High Leaf Starch Landrace

Twenty-nine wheat landraces and twenty-one modern spring wheat cultivars were grown under field conditions and surveyed for starch during grain fill (14 DAF). The average leaf starch value across modern cultivars was 2.60 ± 0.27 µg starch mg^−1^ DW; the cultivar ‘Rescue’ had the highest leaf starch levels (5.99 ± 0.82 µg mg^−1^ DW) (Table S1). Compared to the modern cultivars, the wheat landraces surveyed varied substantially for leaf starch levels and ranged from 0.81–20.34 µg mg^−1^ DW (Table S1). PI 61693 had the highest level (20.34 ± 3.40 µg mg^−1^ DW). Compared to leaf starch levels, seed starch was less variable within landraces and modern cultivars. The overall average seed starch values for all landraces were 73.6 ± 1.0% seed DW at 14 DAF (and modern cultivars had an average of 73.4 ± 2.5%) (Table S1). For the purpose of this study, attention was focused on the landrace with the highest leaf starch levels, PI 61693. A benefit of this approach was that PI 61693 had previously been crossed to ‘Berkut’, a low leaf starch genotype, and a recombinant inbred line mapping population was developed and genotyped [33,34].

### 2.2. Starch Breakdown Is Not Impaired in PI 61693

**Figure 1:**
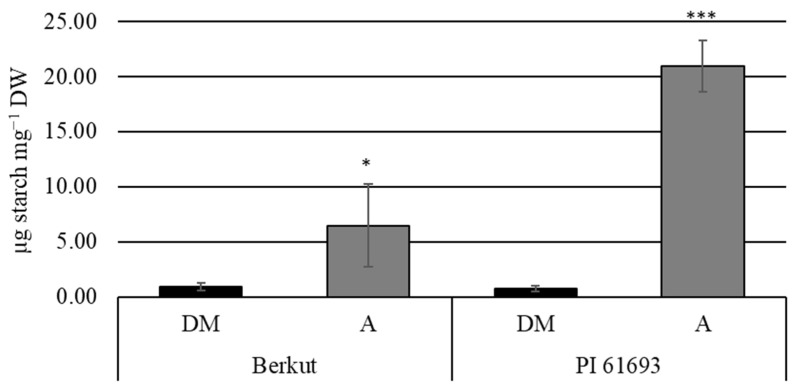
Starch levels in flag leaves collected at 14 DAF in the dark morning (DM; one hour prior to lights on) and mid-afternoon (A) with n = 6 of greenhouse-grown plants. *, *** represent significance at *p*-values ≤ 0.05 and 0.001, respectively, between A and DM measurements within each genotype from one-tailed, equal variance *t*-tests with the hypothesis that starch would be nearly depleted in DM samples.

To examine whether increased leaf starch levels in PI 61693 were consistent with a *starch excess* (*sex*) mutation, flag leaves were collected at 14 DAF one hour prior to lights on and at 4:00 PM, when transitory starch levels should be high. Starch levels were analyzed for Berkut and PI 61693. For both genotypes, starch levels were high at the end of the afternoon and nearly depleted at the end of the dark period (Figure 1), demonstrating that increased starch levels were not the result of changes in starch breakdown.

### 2.3. RIL Starch Frequency Distribution

**Figure 2:**
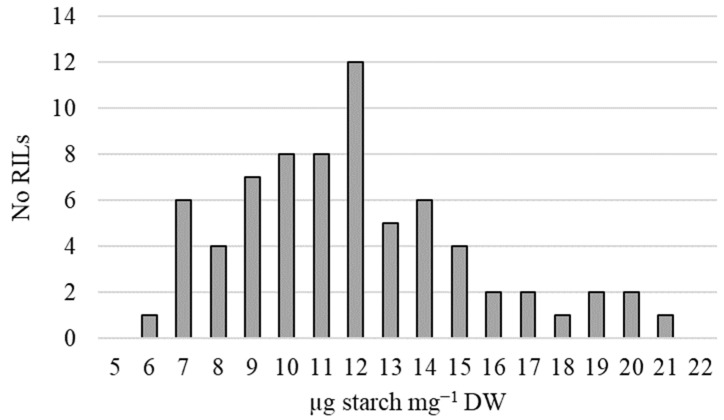
Distribution of leaf starch values from RIL flag leaves at 14 DAF combined over the 2020 and 2021 growing seasons. Collection occurred at mid-afternoon with n = 3 for each RIL during the 2020 and 2021 growing seasons. Each rep consisted of a bulk of three flag leaves from individual plants. Leaf starch concentrations for RIL parent plants Berkut (6.0 ± 0.8 µg mg^−1^ DW) and PI 61693 (23.7 ± 4.8 µg mg^−1^ DW) are not included within the distribution.

Leaf starch at grain fill was quantified in a Berkut × PI 61693 NAM population [33]. Leaf starch values were measured in leaves collected during both the 2020 and 2021 field seasons. RIL starch values fell within a range of 8.6–32.7 µg mg^−1^ DW during the 2020 growing season and 1.7–14.0 in 2021 (Table 1). When compared across years, starch values had a correlation coefficient of 0.25 (*p*-value = 0.03, Table 1). The combined RIL averages were in the range of 5.5–20.4 µg mg^−1^ DW (Figure 2). Starch values were distributed such that PI 61693 values were located near the high end of the distribution curve, whereas Berkut values were located near the bottom. The high starch parent, PI 61693, had a combined average leaf starch value of 23.7 ± 4.8 µg mg^−1^ DW, whereas the low starch parent Berkut had an average leaf starch value of 6.0 ± 0.8 µg mg^−1^ DW (Table 2).

### 2.4. Plant Productivity Is Negatively Correlated to Leaf Starch at Grain Fill in the Berkut × PI 61693 RIL Population

RIL parents Berkut and PI 61693 field data are summarized in Table 2. Landrace PI 61693 had very high levels of leaf starch yet flowered ~4 days earlier than Berkut. In the Berkut × PI 61693-derived RIL population, days to anthesis ranged from 61.0–66.0 days in 2020 and 58.0–67.3 days in 2022 (Table 1). Combined data for individual RILs are presented in Table S2. Due to the strong correlations observed for yield traits between the 2020 and 2021 growing seasons (Table 1), data was combined and correlation analysis was used to examine the relationship between growth parameters. In this NAM population, flag leaf starch at grain fill was negatively correlated to every plant growth and yield trait measured except for seed protein, which had a significant positive correlation (Table 3). The negative correlations between leaf starch at grain fill with seed number per plant and with biomass were especially strong with correlation coefficients of −0.38 and −0.39 respectively (*p*-values ≤ 0.001).

### 2.5. QTL Analysis Identifies BobWhite_c5970_731 Marker Associated with Variation for Leaf Starch and Plant Productivity

QTL analysis identified three QTLs of interest for plant productivity traits (Table 4). In 2020, the markers BS00110350_51 and RAC875_c8842_724 were associated with grain number and grain protein, respectively. The marker BobWhite_c5970_731 was associated with days to anthesis, leaf starch, plant biomass, leaf length, grain number, and seed weight per plant during the 2020 field season. Analysis of the 2021 field data did not reveal any associations; however, when 2020 and 2021 data were combined, BobWhite_c5970_731 was again associated with biomass, grain number, and seed weight per plant in this population. This marker was associated with a 20–34% variation for these traits (Table 4). The BobWhite_c5970_731 SNP is located at position Chr7D: 68,417,466 of IWGSC RefSeq v.1, which is within the coding region of *TraesCS7D02G111600*, a well-conserved *Flowering Locus T1* (*FT-D1*) gene.

### 2.6. Transcript Analysis of Candidate Genes Reveals FT-D1 Is Downregulated in Berkut

To identify candidate genes for leaf starch levels associated with BobWhite_c5970_731 on chromosome 7D, the IWGSC RefSeq map v.1 was used to compose a list of genes located upstream and downstream of BobWhite_c5970_731. Chen et al. (2022) identified 35 genes flanking *TraesCS7D02G111600* (*FT-D1*), in which BobWhite_c5970_731 is located. These genes span a region covering 7D: 66,373,919–69,266,821 [35]. We expanded upon these findings by 4× to identify 165 genes located within 7D: 62,106,312–73,743,194 (Table S3). To further evaluate the likelihood of these candidate genes as causal for starch differences, we carried out transcript expression analysis on the RIL parent material. Expression values as well as protein name and putative function can be found in Table S3. Of the 165 candidate genes, 20 had expression levels ≥ 5.0 (Table 5). Of these, three genes stood out as interesting based on expression levels and/or putative gene function. The first is *TraesCS7D02G103500*, a 2Fe-2S ferredoxin-type domain-containing protein that is involved in the photosynthesis electron transport chain. However, there was no difference in expression between Berkut (262.43) and PI 61693 (248.63). The second is *FT-D1*, *TraesCS7D02G111600*. Berkut, which carries the frameshift mutation, had much lower levels of expression compared to PI 61693 at 50.58 vs. 219.66. The third gene of interest is *Starch synthase wSsI-1, TraesCS7D02G117800*. For this genotype, Berkut had a 1.5× increase in expression compared to PI 61693 (42.82 vs. 28.37).

### 2.7. Sequence Differences in FT-D1 (TraesCS7D02G111600) in Berkut vs. PI 61693 RIL Parents

In order to examine *TraesCS7D02G111600* as a candidate gene, DNA from RIL parents was sequenced with D-genome-specific *TraesCS7D02G111600* primers. A single base pair deletion (G) was identified in Berkut 840 base pairs downstream of the translation start codon. This insertion is described by [35] and termed *FT-D1ΔG*. The single base pair mutation is located at IWGSC RefSeq v.1 Chr7D: 68,417,122. This indel segregated with starch and plant productivity within this population (Table S2). Furthermore, in addition to flowering later and lower leaf starch, RILs with the *FT-D1ΔG* allele had significantly higher biomass, seed number, and seed weight per plant compared to those carrying the full-length allele (Table S2).

### 2.8. Free Sugar Levels in Flag Leaves Were Consistent with the Presence of FT-D1ΔG in Low Starch Lines and the Full Length Allele in High Starch Lines

**Figure 3:**
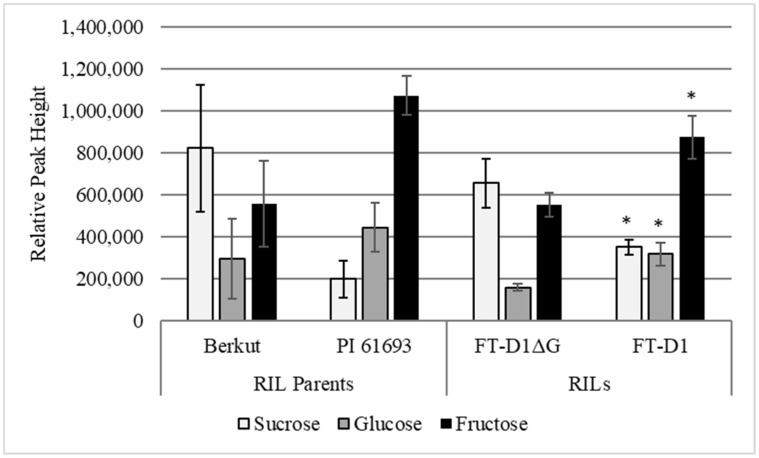
Flag leaf sugars were measured at 14 DAF in RILs segregating for low leaf starch and the FT-D1ΔG allele versus RILs with high leaf starch and the full-length FT-D1 allele (n = 4). RIL parent Berkut carries *FT-D1ΔG*, whereas PI 61693 carries the full-length allele. Metabolite data were obtained via GC-MS/MS analysis and values represent relative peak heights. * represent significance at *p*-values ≤ 0.05, between *FT-D1ΔG* and *FT-D1* genotypes for each sugar using two-tailed, equal variance *t*-tests.

RILS with the highest and lowest leaf starch phenotypes were identified based on combined 2020 and 2021 starch data (n = 15 for each group). From this set, four representative lines were selected such that height was held constant across starch phenotype. KASP assay results confirmed the presence of the single base pair deletion *FT-D1* allele in the low starch phenotypes and the absence of the deletion in the high starch phenotypes. Sucrose, glucose, and fructose all trended in the direction of the parent allele for both high and low starch phenotypes. Low starch lines (*FT-D1ΔG*) had significantly higher levels of sucrose, whereas high starch lines (full-length allele) had significantly higher levels of glucose and fructose (Figure 3).

## 3. Discussion

Transitory leaf starch is an important energy source for plant growth and development [4]. Lack of leaf starch is detrimental to cereal growth and yield [18], whereas upregulated leaf starch metabolism has led to enhanced plant growth and yield in transgenic cereal populations [20,21,22]. Results from these studies indicate that cereal yield may be enhanced via the development of selective breeding methods for specific leaf starch levels. To move toward that goal, this study examined starch levels from an array of modern wheat cultivars and landraces to identify natural sources of flag leaf starch variation.

In this study, 21 modern cultivars and 29 landraces were surveyed for flag leaf starch levels at 14 DAF. This stage was selected since transgenic rice plants overexpressing leaf starch biosynthesis had the greatest difference in leaf starch compared to WT sister lines at this stage of development [22]. This is also a critical stage where energy in source tissue is actively mobilized to sink organs. Of the modern cultivars surveyed, ‘Rescue’ had the highest level of flag leaf starch (5.99 ± 0.82 µg mg^−1^ DW), which was 2.3× that of the average for modern cultivars (2.60 ± 0.27 µg mg^−1^ DW) (Table S1). Interestingly, Sawtana, Fortuna, and Newana also had higher than average levels of leaf starch (Table S1); these cultivars include Rescue in their pedigrees, indicating that there may be a genetic component to leaf starch phenotype in these varieties.

In order to determine whether the high starch phenotype observed in PI 61693 was due to inefficient starch breakdown during the dark period, leaves were collected at the end of the dark period and compared to leaves collected during the late afternoon, when starch levels should be high (Figure 1). It is well documented that the inability to break down leaf starch, as seen in *Arabidopsis starch excess 1* (*sex1*) mutants, results in stunted, low-yielding plants [36,37]. Furthermore, mutations that lead to leaf starch accumulation resulting from lack of carbohydrate export have also been shown to be detrimental to plant growth and yield in maize [38,39,40]. Here, it was determined that elevated leaf starch levels were not due to the inability to break down starch at night. Starch levels were nearly depleted at the end of the dark period in both RIL parents, PI 61693 and Berkut (Figure 1).

Leaf starch at 14 DAF was measured across two field seasons in the Berkut × PI 61693 RIL population [33]. As expected, PI 61693 segregated at the high end of the distribution curve, while the low starch parent, Berkut, was located near the bottom of all detected values (Figure 2, Table 3). Results from transgenic studies discussed above indicate that improved leaf starch biosynthesis is beneficial to plant growth. Therefore, at the onset of this project, the working hypothesis was that increased leaf starch biosynthesis was likely beneficial to plant growth. However, in this population, there was a strong negative correlation between leaf starch and almost all productivity traits (Table 4).

A large amount of variation in plant height and flowering date was observed in this population (Table 1), despite selection pressure for reduced height (*Rht*) and day length insensitivity (*Ppd*) during RIL development [33]. Variation for these two important traits translated to high levels of significance for correlations between all plant-growth-related traits in this population (Table 3). Regardless of this variation, QTL analysis identified the marker BobWhite_c5970_731 to be associated with 20–34% variation for days to anthesis, leaf starch, plant biomass, and grain yield (Table 4). Although this QTL was not identified during the 2021 field season, it was identified in 2020 and when the 2020 and 2021 growing season data were combined. This likely reflects the low number of RILs in this population (n = 68) and differences between growing years. While the 2021 growing season received an extra 5 cm of precipitation compared to 2020, it was also 5 °C warmer during June and July relative to 2019 and 2020 (Table 6). During both the 2020 and 2021 growing seasons, there was a strong negative correlation (*p*-values ≤ 0.001) between leaf starch at grain fill and plant productivity (Table 3). The lower starch values observed in 2021 may reflect a downregulation of starch by heat stress [26]. As transitory leaf starch is a product of photosynthesis, environmental conditions such as temperature, cloud cover, and humidity influence stomatal conductance and contribute to differences between data sets [41].

A list of candidate genes located upstream and downstream of BobWhite_c5970_731 was created and transcript expression analysis was performed. Based on putative protein function, the immediate gene of interest behind leaf starch differences at grain fill is the starch synthase gene *wSsI-1* (*TraesCS7D02G117800*). Interestingly, Berkut had a 1.5× increase in expression over PI 61693. Considering that PI 61693 had much higher levels of leaf starch, this came as a surprise. Further evaluation of *wSsI-1* and experiments designed to specifically address the causal differences in starch between RIL parents will be needed.

Based on expression levels, *TraesCS7D02G111600*, encoding a flowering locus T protein, is the most likely candidate gene for plant growth differences within this population. There was a 4.34× increase in expression of this gene in RIL parent PI 61693 (219.66) over Berkut (50.58) in flag leaves at grain fill. Furthermore, the BobWhite_c5970_731 SNP marker is located within the coding region of *TraesCS7D02G111600*. *TraesCS7D02G111600* is orthologous to the *Arabidopsis thaliana* gene *FLOWERING LOCUS T* (*FT*) [42]. In wheat, this gene is known as *FT1* and sometimes *Vrn-3*. *FT1* is a well-conserved florigen gene across flowering plants that encodes a phosphatidylethanolamine-binding (PEBP) transcription factor involved in both the photoperiod response and vernalization pathways [43].

To examine the likelihood that differences in the *FT-D1* sequence were responsible for the phenotypes observed in this RIL population, the *FT-D1* coding regions from RIL parents PI 61693 and Berkut were sequenced. A single base G deletion in the third exon of the Berkut allele was identified. These two haplotypes were first described in [44] and are reported as partial cds GenBank accessions EF428113 and EF428114 called *FTD-h1* and *FTD-h2*. Since then, the entire *FT-D1* gene, including the upstream and downstream sequences, was published in a study by Chen et al. (2022), in which the authors also identified the same deletion (termed *FT-D1ΔG*) and determined that *FT-D1ΔG* was responsible for delayed flowering and increased spikelet number in a RIL population [35]. Chen et al. (2022) further validated their findings with CRISPR/Cas9 *FT-D1* knockout mutants and demonstrated that mutant lines flowered later and had significantly increased spikelet numbers. In this Berkut/PI 61693 population, Berkut (carrying *FT-D1ΔG*) flowered ~ four days later than PI 61693 and had much lower leaf starch at grain fill (Table 2). Furthermore, RILs homozygous for the *FT-D1ΔG* allele had significantly lower leaf starch, yet significantly higher biomass, seed number, and seed weight per plant (Table S2). These findings support those published by Chen et al. (2022) [35]. More research is needed to determine whether leaf starch differences in this population are directly linked to *FT-D1*, are from another gene segregating with *FT-D1*, or are from a downstream pathway mediated by *FT-D1*.

*FT1* is involved in many aspects of plant development. In non-cereal crops, *FT1* homologs have been shown to promote bulb formation in onions [45] and are involved with pod number and seeds per plant in soybeans [46]. The rice homolog, HEADING DATE 3a protein (HD3a), is transported to the shoot apical meristem, where it forms an activation complex to promote the expression of genes involved in the transition from vegetative to reproductive growth [47]. Further research in rice demonstrated that Hd3a protein accumulates in the axillary meristem to promote branching [48]. Studies in wheat have shown that FT1 interacts with TB1 to influence tiller formation [49] and there has been a growing body of evidence linking *FT1* with wheat spikelet formation [35,49,50,51,52].

Although the underlying cause of leaf starch differences in the Berkut × PI 61693 RIL population remains unknown, there was a strong negative correlation between leaf starch at grain fill with flowering date and most plant productivity traits (Table 3). This inverse relationship suggests that overall carbon metabolism may be more efficient in plants with lower levels of leaf starch in this population. To examine this hypothesis, metabolites were extracted from five RILs carrying the *FT-D1ΔG* allele and five carrying the full-length *FT-D1G* allele. In these plants, it was found that levels of glucose and fructose were lower in plants carrying *FT-D1ΔG* compared to those with the intact *FT-D1G* allele, whereas sucrose levels were higher (Figure 3). It is possible that *FT-D1* variation is directly responsible for the changes in carbon metabolism observed in this study, or from a linked gene segregating with *FT-D1*. Alternatively, these differences in simple sugars may reflect larger differences in rates of senescence resulting from *FT-D1* allele presence. Although rates of senescence and days to maturity were not measured in this study, Chen et al. (2022) reported that this single base pair deletion has an even greater effect on promoting physiological maturity than the heading date in a winter wheat population grown in China [35]. Wang et al. (2015) showed that sucrose levels peak near 10 DAF and declined sharply throughout the rest of the grain fill [53]. In this study, flag leaf samples were collected at 14 DAF. If the time to maturity is altered in lines carrying the *FT-D1ΔG* allele, levels of flag leaf carbon metabolites may reflect that difference.

It is possible that changes in starch and sugar metabolites reflect differences in sucrose metabolism tied to *FT-D1*. Significant changes in sucrose levels (Figure 3) are interesting because sucrose is the major long-distance photosynthate transport molecule [54]. Furthermore, sucrose acts as a signaling molecule for flowering in *Arabidopsis thaliana* [55,56]. However, in this study, increased sucrose is associated with later flowering. An alternative hypothesis is that the altered carbohydrate levels are simply an indicator of sugar transport changes. However, any sugar transporters identified within the candidate region had very low (≤5.0) levels of expression (Table S3).

Research carried out in potatoes (*Solanum tuberosum*) supports the hypothesis that sucrose transport may be disrupted by *FT1* allelic variation. In potatoes, the FT-like protein StSP6A induces tuberization, while another FT-like protein induces flowering [57]. Tuberization is induced by sucrose in vitro [58,59]. Abelanda et al. (2019) investigated the link between sucrose metabolism and *StSP6A* expression and found that sucrose triggers *StSP6A* tuber-specific expression [60]. They demonstrated protein–protein interactions between StSWEET11 and StSP6A at the cytosolic face of the plasma membrane. The implication of this is that carbon allocation throughout the plant may be altered via a similar interaction. Therefore, FT-D1 may interact with a sugar transporter influencing flag leaf sugar export, which could affect flag leaf starch levels.

A final possible explanation for starch differences between our RIL parents is that FT-D1 interacts with a transcription factor involved in carbon metabolism such as one belonging to the WRKY family. Wang et al. (2023) examined the transcriptome of winter wheat during vernalization and found that many transcription factors were significantly increased in expression during vernalization; among these were 129 WRKY transcripts [61]. An Arabidopsis study found that AtWRKY75 is a positive regulator of flowering initiation and binds to the *FT* promoter [62]. There is also evidence that WRKY transcription factors regulate leaf starch metabolism [29,63].

It is well established that *FT1* interacts with earliness genes within the photoperiod and vernalization pathways. Epistatic interactions are a likely explanation for why QTL encompassing *FT-D1* are not always picked up on association mapping panels even when *FT-D1* polymorphism is known to exist within a population [64,65]. In a wheat study in which epistatic interactions for early flowering loci were examined, it was found that *vrn-A1* > *VRN-B1* > *vrn-D3* (aka *FT-1D*) > *PPD-D1* for intensity of effect [66]. As was observed in this study, the G × E effect also plays an important role in whether *FT1* QTL will be identified in any individual environment (Table 4 and Table 6). Interestingly, Kiss et al. (2017) reported that the expression of *Vrn-3* (*FT1*) is repressed with increased thermal time [67]. As noted above, the 2021 field season was an average of 5 °C warmer for June and July compared to 2019 and 2020. This provides a reasonable hypothesis for why no QTL associated with *FT1* was found using the 2021 field season data.

It was originally hypothesized that higher leaf starch would correlate with plant productivity in wheat. This study demonstrated the contrary, likely confounded by the large difference in flowering time caused by the *FT-D1ΔG* allele in conjunction with the experimental design. Studies on photoperiod genes demonstrated that earlier flowering times are beneficial to yield in hot and dry growing regions, while later flowering is favorable under cooler and wetter conditions [68]. This is supported by Dreisigacker et al. (2021), who showed wheat yield under irrigation was highest for varieties with delayed flowering [69]. Delayed flowering allows for increased biomass accumulation prior to anthesis and promotes the development of a larger seed head. So long as these larger plants are supported by continuous water delivery during gain fill, their yield is increased appropriately.

In hotter and/or drier environments, with no additional irrigation, earlier flowering is beneficial to yield because anthesis occurs before high heat can decrease pollen germination and water is retained in the soil for grain fill [70,71]. All field experiments in this study were supported by irrigation, thus yield was favored by the considerably later (4 days) flowering allele. However, the high starch allele was associated with the earlier flowering phenotype. The average time from emergence to anthesis for spring wheat is between 40 and 50 days, making the 4-day difference in flowering time observed in this experiment very significant. It could be that the extreme difference in flowering time overcame any potential benefit to yield afforded by higher leaf starch during grain fill. Further studies will be necessary to separate leaf starch from flowering time to determine the potential impact of leaf starch without this interaction. This can be achieved by identifying other QTLs for leaf starch that are not also associated with flowering time, and by carrying out further field trials with this RIL population designed to address heat and/or drought stress.

**Figure 4:**
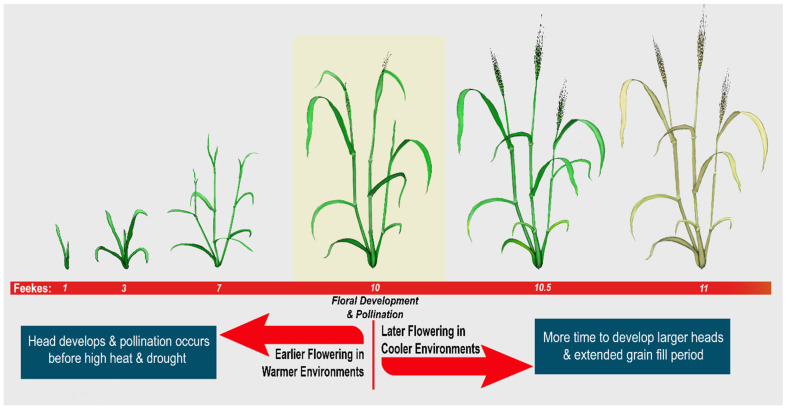
Model for Flowering Time Breeding Efforts Based on Environment. Warmer, dryer environments (dryland) see increased yield with earlier flowering time while cooler, wetter environments (irrigated) see increased yield with later flowering time.

The impact of *FT-D1ΔG* on flowering time has major implications for climate change. Control over the timing of floral development at the genetic level can be exploited to adapt wheat cultivars to the direction of temperature and precipitation changes in the target environment. Here, we proposed a generalized model for adapting spring wheat flowering time to different environmental regions for enhanced yield (Figure 4). In growing regions that are becoming warmer, with an extended frost-free day timeline, a longer growing season can be beneficial to irrigated wheat where flowering time has been pushed back. Conversely, in growing regions that are seeing a reduction in rainfall during grain fill, and where irrigation is not practical, wheat yields benefit from an earlier flowering time.

In addition to the *FT-D1* polymorphism described here, *FT-A1* and *FT-B1* polymorphisms have been reported [35,42,44,72]. Identifying allelic variants of *FT1* within each wheat genome-specific copy and other earliness genes within breeding populations will allow for pyramiding for target flowering time and maturity to target growing regions. Research utilizing heterogeneous inbred families (HIFs) that vary for one or more *FT-1* alleles will provide insight into metabolic mechanisms and the degree to which *FT-1* impacts wheat plant growth and productivity in different genetic backgrounds.

## 4. Materials and Methods

### 4.1. Starch Survey Plant Material

Twenty-one spring wheat cultivars were released between 1910 and 2005 [73] and 29 landraces [33] were field-grown in 2019 at the Arthur H. Post Farm near Bozeman, MT, and surveyed for native levels of flag leaf and seed starch at grain fill. Origins of plant material may be found in Supplementary Table S1. The landraces were collected from the continents of Africa (7), Asia (13), Europe (4), and South America (5) and are described in Blake et al. (2019) [33].

### 4.2. Recombinant Inbred Line (RIL) Plant Material

The recombinant inbred line population was developed by crossing landrace parent PI 61693 to the CIMMYT spring wheat cultivar ‘Berkut’ (Irene/Babax//Pastor, released in 2002) and consisted of 68 F_5_ derived recombinant inbred lines (RILs). The landrace PI 61693 originated in Malawi and has recently been integrated into the USDA WheatCAP Coordinated Agricultural Project, where it is described by Blake et al. (2019) and Jordan et al. (2018) [33,34]. The RIL population used in this study was one of the RIL populations from the NAM population described by Blake et al. (2019) [33]. During development, selection pressure for height and early flowering was applied for each generation. Field trials of RILS were planted with F_5_-derived F_9_ (F_5:9_) seed in 2020 and F_5_-derived F_10_ (F_5:10_) seed in 2021.

### 4.3. Field Trial Setup

For each field trial, the seed was planted at the Arthur H. Post Farm near Bozeman, MT in early May once the average soil temperature exceeded 4.4 °C. The soil was tilled and was seed sown with a disk seeder following a fallow rotation from wheat. Yield trials were carried out in a complete randomized block design with three replications. Space plant density consisted of 3m rows with 15 seeds planted per row. At Feekes stage 1, rows were thinned to 10 evenly spaced plants. Weed control was managed each spring with the application of herbicide at the beginning of June. In 2019, the field was sprayed with Huskie Complete Herbidice (Bayer CropScience, LP, St. Louis, MO, USA) at a rate of 1.10 L ha^−1^. The 2020 field was sprayed with a combination of herbicides Affinity (4.7 mL ha^−1^; FMC Corporation, Philadelphia, PA, USA), MCP ester 4 (0.58 L ha^−1^; Loveland Products, Inc., Loveland, CO, USA), Discover (0.58 L ha^−1^; Syngenta Crop Protection, Greensboro, NC, USA), and the fungicide Propi Star EC (0.29 L ha^−1^; Albaugh, LLC, Ankeny, IA, USA). The 2021 field was sprayed with 1.75 L ha^−1^ Vendetta (Wilbur-Ellis Co., Fresno, CA, USA) and 0.077 L ha^−1^ Parity (Tenkoz Inc. Alpharetta, GA, USA). Irrigation occurred one week prior to and one week after anthesis. Three representative plants were harvested per row and bundled. Bundles were dried and biomass was recorded. Bundles were threshed using a single plant thresher. Yield parameters measured included days to flowering, plant height and tiller number at maturity, seed weight per plant, and kernel characteristics. Grain protein and moisture were measured using a Foss Infratec 1241 Machine in which near-infrared absorption was compared to control reference values to determine protein [74].

### 4.4. Yield Trial Weather

Here, we define a field season at the Arthur H. Post Farm as running from 1 May to 31 August. During the 2019 field season, the Arthur H. Post Farm received 18.62 cm of precipitation. The lowest air temperature of −4.2 °C was recorded on 1 May, whereas the highest was recorded on 23 July at 32.3 °C. During the 2020 field season, the research center received 13.44 cm of precipitation. The lowest air temperature was recorded on 8 May at −0.03 °C. The highest recorded air temperature was 33.4 °C on 2 August. During the 2021 field season, the research center received 17.0 cm of precipitation. The lowest air temperature of −4.1 °C was recorded on 22 May while the highest air temperature was recorded on 18 July at 35.8 °C. Average temperatures for May-August are reported in Table 6.

### 4.5. Tissue Collection

Sampling occurred 14 days after flowering (DAF) +/− one day at 4:00 PM. Three replicates were collected, in which each biological replicate consisted of a single row and was the bulk composite of three flag leaves or heads harvested from individual plants. For the original screen of modern cultivars and landraces, entire flag leaves and heads were collected. The tissue was immediately frozen in liquid N_2._ Heads were threshed while remaining frozen and leaf and seed tissue was ground to a fine powder. Frozen tissue was allocated for starch quantification and molecular experiments. Sampling of the RIL population was carried out as described above, though heads were not collected.

### 4.6. Starch Quantification

Starch assays were carried out by Oiestad et al. (2019) [63]. Starch was extracted according to Smith and Zeeman (2006) from 10 mg dry weight (DW) samples [75]. Briefly, free glucose was removed from ~40 mg fresh weight (FW) ground powder with the addition of 80% EtOH and heated to 80 °C for 3 min. Ethanol supernatants were discarded, and this step was repeated twice. Pellets were dried and DW (~10 mg) was recorded. Pellets were suspended in 300 µL of 100 mM sodium acetate, pH 5.5. Leaf starch samples were digested with 0.06 U α-amylase and 0.18 U amyloglucosidase mg^−1^ DW. Seed starch samples were digested with 0.2 U α-amylase and 0.6 U amyloglucosidase mg^−1^ DW. Samples were assayed for starch according to Rӧsti et al. (2007), in which the change in NADPH (stoichiometric with D-glucose) was observed by the increase in absorbance at 340 nm [76]. Starch concentrations were determined by referencing a standard curve prepared from known amounts of purified wheat seed starch.

To examine leaf starch at the end of the dark period, plants were grown in a greenhouse with conditions consisting of a 16 h photoperiod with 22 °C day and 18 °C night temperatures. Flowering dates were recorded, and flag leaves were collected at 14 DAF +/− one day at one hour prior to lights on (dark morning, DM) and at 4:00 PM (mid-afternoon, A). Leaves were immediately frozen in liquid N_2_ and ground to a fine powder. Starch was extracted and assayed as described above. For comparisons between dark morning (DM) and afternoon (A) samples, one-tailed, equal variance t-tests were used to determine the significance between starch level harvested at DM vs. A with the hypothesis that starch would be depleted in DM samples.

### 4.7. Metabolite Analysis

RILs were selected for metabolomic analysis by sorting according to leaf starch level. The RILs with the highest starch values and lowest values that also had average height were selected with n = 4 for high and low starch phenotypes. The presence of the full-length *FT-1D* allele was confirmed for the high starch lines and the presence of the *FT-D1ΔG* allele was confirmed for the low starch lines. Samples were collected as described above with three biological replicates for each RIL. Frozen tissue was ground to a fine powder, freeze-dried, and aliquoted into 7 mg DW samples. Metabolites were extracted as per Schmidt et al. (2011), where 350 µL methanol (60 °C) was added to each sample, followed by incubation at 60 °C for 10 min [77]. Samples were vortexed and placed in a sonicating water bath for 10 min. Chloroform (350 µL) was added, and samples were vortexed followed by the addition of 300 µL water. Samples were again vortexed followed by centrifugation at full speed for 5 min. Polar fractions were transferred to GC-MS glass vials (150 µL per 7 mg DW) and dried in a speed-vac concentrator. Sample analysis was carried out using an Agilent 6890 gas chromatograph (Agilent Technologies, Santa Clara, CA, USA). Protocols laid out in Fiehn et al. (2008) were followed for data acquisition, metabolite identification, and sample normalization [78].

### 4.8. Transcript Analysis

Samples were collected as described above for the original starch screen 14 days after flowering (DAF), +/− one day, at 4:00 PM. Samples were collected from 10 landraces and 10 modern cultivars, representing low, mid, and high levels of leaf starch from the 2019 starch survey. Supplemental Table S1 indicates inclusion in this experiment. Pedigree and country of origin were also given consideration. From each replicate, 30 mg of frozen powder was combined into a single sample, such that samples consisted of a bulk of nine plants for each genotype.

RNA was extracted using an RNeasy Plant Mini Kit (QIAGeN, Germantown, MD, USA) according to the manufacturer’s instructions. RNA integrity was examined using an Agilent 2100 Bioanalyzer and all samples had RINs ≥ 6.8. Samples were sent to LC Sciences (Houston, TX, USA) for RNA-seq analysis. A total of 1 µg RNA was used to create cDNA libraries, and amplicons were sequenced as paired-end, 150 bp reads using an Illumina High Scan-SQ platform with a depth of 6 GB for each sample.

Twenty leaf and twenty seed samples were sent in for sequencing. The average raw reads across the 40 samples were 45,946,206 ± 851,410. LC Sciences used Cutadapt [79] and perl scripts in-house to remove reads that contained adaptor contamination, low-quality bases, and undetermined bases. Sequence quality was then verified using FastQC (http://www.bioinformatics.babraham.ac.uk/projects/fastqc/, accessed on 3 December 2019). The average total clean reads across were 42,180,475 ± 840 567. Data was assembled and analyzed using Ngen and ArrayStar within the DNASTAR Lasergene v 17.6.2.9 software suite (DNASTAR, Madison, WI, USA). Reads were aligned to the *Triticum astivum* genome (iwgsc_refseqv1.0) within the DNA-star software suite using RPKM normalization. All other settings were left at default.

### 4.9. Data Analysis

Correlation analysis and creation of histograms were carried out using the Data Analysis Toolpak in Microsoft Excel, accessed 7 October 2021. The *p*-values for correlation coefficients were determined using a *p*-value calculator for correlation coefficients [80] with n = 68. Analysis of variance was carried out for each response variable using a randomized split-plot model combined over replications in R 4.1.2 [81] with the car [82] and emmeans [83] packages.

### 4.10. Genotyping, Linkage Map Construction, and QTL Analysis

Genotyping was previously carried out using the Illumina 90K iSelect assay [84] and is described in Jordan et al. (2018) [34]. During analysis, monomorphic markers, markers with >10% missing genotypes, and markers with significantly distorted segregation ratios were discarded. A genetic map was created using protocols described by Varella et al. (2019) [85] using the R/qtl and R/ASMap packages in R [86,87]. Co-segregating markers were discarded and polymorphic markers were excluded if they had >25% missing data or showed significant Mendelian segregation distortion (Chi-square test, *p* < 1.0 × 10^−7^, df = 1).

### 4.11. Amplification of FT-D1 (TraesCS7D02G111600)

Screening for *FT-D1* polymorphism between parental lines was assessed with two sets of primers. A 2284-bp fragment of *FT-D1* was amplified using forward primer 5′ GATCCATCCATCGGTCTC 3′ and reverse primer 5′ GCTGAATGACAAGAGCTGA 3′, which amplified all exons in the gene. The amplicon was sequenced using both the forward and reverse primer, sequencing both exons and ignoring the large intron between them. The PCR amplification reaction was as follows: one cycle of 96 °C for 5 min, 40 cycles of 96 °C for 30 s, 57 °C for 30 s, 72 °C for 180 s, and one cycle of 72 °C for 7 min with a final hold at 4 °C.

A single base pair deletion (G) was found in Berkut at the 840th base pair. This deletion leads to a frameshift mutation in the amino acid sequence and was first described by Bonnin et al. (2008) [44]. This mutation was termed *FT-D1ΔG* and its effect on wheat plant development was recently characterized in Chen et al. (2022) [35]. A competitive allele-specific PCR (KASP) assay was developed for the purposes of genotyping the RIL population for *FT-D1*. The full-length allele, from PI 61693, was amplified with the reverse primer and FAM tag 5′ GAAGGTGACCAAGTTCATGCTGAAGCGATGGATCCCC 3′, while the *FT-D1ΔG* allele, from Berkut, was amplified with the reverse primer and HEX tag 5′ GAAGGTCGGAGTCAACGGATTGAAGCGATGGATCCCA 3′. A common forward primer, 5′ GGTACAACTGGTGCATCC 3′, was utilized for the assay. The assay produced a 76-bp product that identified the FAM tag for full-length alleles and the HEX tag for alleles containing the deletion. The assay was performed on a BioRad CFX Opus 96 Real-Time qPCR system with the following reaction protocol: one cycle of 94 °C for 15 min, 10 cycles of 94 °C for 20 s, 61 °C for 60 s with a 0.6 °C decrease every cycle, 29 cycles of 94 °C for 20 s, 55 °C for 60 s, 30 °C for 20 s, followed by a single cycle of 30 °C for 5 min with a final hold at 4 °C.

## Figures and Tables

**Table 1:** Growth and yield of recombinant inbred lines (RILs) derived from a Berkut × PI 61693 cross-grown during the 2020 and 2021 growing seasons ^a^.

	Days to Anthesis	Leaf Starch (µg mg^−1^ DW)	Height ^b^ (cm)	Biomass ^b^ (g)	Seed Wt ^b^ (g)	ISW ^b^ (mg)	Seed No ^b^	Seed Protein ^c^	HI ^c^
**2020**									
Range	61.0–66.0	4.26–32.7	55.9–92.7	26.5–146	8.9–46.5	31.5–50.5	226–1,099	12.1–18.6	0.28–0.37
Mean	62.8	17.3	73.7	66.1	21.6	40.5	540	14.4	0.33
*F*	2.60 ***	1.95 ***	4.50 ***	4.01 ***	3.63 ***	3.63 ***	3.58 ***	7.74 ***	2.57 ***
**2021**									
Range	58.0–67.3	1.83–16.3	48.0–94.0	24.3–109	4.8–33.5	22.7–42.3	168–937	12.3–16.2	0.18–0.40
Mean	61.5	5.2	65.7	58.6	17.8	32.4	552	14.0	0.30
*F*	5.83 ***	4.10 ***	7.70	4.79 ***	3.70 ***	3.70 ***	3.37 ***	5.32 ***	1.04
**Combined**									
Range	59.7–66.0	5.5–20.4	51.9–87.9	25.4–126	6.9–40.0	27.7–46.4	202–999	12.3–17.1	0.24–0.35
Mean	62.2	11.3	69.7	62.4	19.7	36.4	543.7	14.2	0.32
*F*	5.17 ***	2.00 ***	9.32 ***	5.16 ***	4.79 ***	9.34 ***	7.09 ***	9.29 ***	1.86 ***
**2020 v 2021**									
*r*	0.56 ***	0.25 *	0.71 ***	0.58 ***	0.57 ***	0.72 ***	0.58 ***	0.75 ***	0.21 ***

^a^ Leaf starch was measured in flag leaves collected mid-afternoon at 14 DAF with n = 3. ^b^ Three plants per plot were harvested and growth parameters are reported as the average of three plants with n = 2 for each genotype. Days to anthesis is the average of all three plots (n = 3). ^c^ ISW represents individual seed weight and HI represents harvest index. Protein is based on 12% moisture. *, *** represent significance at *p*-values ≤ 0.05, and 0.001, respectively.

**Table 2:** Growth and yield parameters for RIL parents PI 61693 and Berkut for the 2020 and 2021 field seasons ^a^.

	Days to Anthesis	Leaf Starch (µg mg^−1^ DW)	Height (cm)	Biomass (g)	Seed Wt (g)	ISW (mg)	Seed No	Seed Protein	Harvest Index
**2020**									
Berkut	66.0 ± 2.1	4.3 ± 1.1	74.9 ± 1.3	67.3 ± 22.6	21.2 ± 7.5	39.4 ± 1.4	545 ± 208	13.4 ± 1.3	0.31 ± 0.01
PI 61693	62.0 ± 0.6	31.2 ± 7.7	72.4 ± 1.3	37.3 ± 2.6	12.2 ± 1.4	40.1 ± 0.6	304 ± 30	14.2 ± 0.1	0.33 ± 0.01
*p*-Value	0.14	0.07	0.29	0.32	0.36	0.67	0.37	0.62	0.46
**2021**									
Berkut	63.0 ± 0.6	7.2 ± 0.2	63.7 ± 1.2	50.6 ± 2.8	16.8 ± 1.0	34.9 ± 0.5	482 ± 36	15.0 ± 0.4	0.33 ± 0.0
PI 61693	59.3 ± 0.3	16.3 ± 0.9	65.3 ± 4.5	61.9 ± 0.8	20.5 ± 1.0	35.9 ± 0.4	572 ± 35	15.1 ± 0.3	0.33 ± 0.01
*p*-value	0.01	0.001	0.74	0.06	0.12	0.28	0.21	0.84	0.89
**Combined**									
Berkut	64.5 ± 1.5	6.0 ± 0.8	68.2 ± 2.9	58.9 ± 10.5	19.0 ± 3.3	37.1 ± 1.4	513 ± 88	14.2 ± 0.7	0.32 ± 0.01
PI 61693	60.7 ± 0.7	23.7 ± 4.8	68.2 ± 3.0	49.6 ± 7.1	16.3 ± 2.5	38.0 ± 1.3	438 ± 80	14.6 ± 0.3	0.33 ± 0.01
*p*-Value	0.02	0.01	1.0	0.49	0.55	0.67	0.55	0.60	0.57

^a^ Values represent average ± SE with n = 2 for each genotype except days to anthesis and leaf starch where n = 3. Leaf starch was measured in flag leaves collected mid-afternoon at 14 DAF. Growth parameters such as biomass and seed weight are reported on an individual plant basis. ISW represents individual seed weight. Protein is based on 12% moisture. *p*-Values are from 2-tailed *t*-tests with equal variance.

**Table 3:** Correlation coefficients for plant growth and yield for a Berkut × PI 61693 RIL population grown under irrigated field conditions combined over the 2020 and 2021 field seasons ^a^.

	Days to Anthesis	Leaf Starch	Height	Biomass	Seed Wt	Individual Seed Weight	Seed No	Seed Protein	Harvest Index
Days to Anthesis	--								
Leaf Starch	−0.25 *	--							
Height	0.38 ***	−0.13	--						
Biomass	0.60 ***	−0.39 ***	0.65 ***	--					
Seed Wt	0.57 ***	−0.40 ***	0.58 ***	0.99 ***	--				
Individual Seed Weight	0.10	-0.01	0.45 ***	0.19	0.13	--			
Seed No	0.51 ***	−0.38 ***	0.41 ***	0.90 ***	0.94 ***	-0.19	--		
Seed Protein	−0.11	0.24 *	0.02	−0.27 **	−0.35 **	0.23 *	−0.41 ***	--	
Harvest Index	−0.01	−0.24 *	−0.19	0.21	0.33 **	−0.16	0.39 ***	−0.51 ***	--

^a^ Plants were grown under space plant density and each genotype was replicated within a complete randomized block design. *, **, *** indicate 2-tailed probability at *p*-values ≤ 0.05, 0.01, 0.001, respectively.

**Table 4:** Quantitative trait loci (QTL) identified for plant productivity and leaf starch at grain fill in a Berkut × PI 61693 RIL population.

Trait	Peak Marker	Chr	QTL Position (cM)	LOD	Source	Effect	Percent Effect
**2020**							
Days to Anthesis	BobWhite_c5970_731	7D	76	4.6	PI 61693	Earlier	26.6
Leaf Starch	BobWhite_c5970_731	7D	77	3.9	PI 61693	Increase	22.9
Biomass	BobWhite_c5970_731	7D	76	6.3	PI 61693	Decrease	34.2
Seed No	BobWhite_c5970_731	7D	79	6	PI 61693	Decrease	20.6
Seed No	BS00110350_51	3A	111	3.8	PI 61693	Decrease	9.9
Seed Weight	BobWhite_c5970_731	7D	76	5.7	PI 61693	Decrease	31.5
Protein	RAC875_c8842_724	7D	37	4.2	PI 61693	Increase	24.3
**2021**							
None detected							
**Combined**							
Biomass	BobWhite_c5970_731	7D	76	4.6	PI 61693	Decrease	26.4
Seed No	BobWhite_c5970_731	7D	77	4.8	PI 61693	Decrease	27.3
Seed Weight	BobWhite_c5970_731	7D	76	4.3	PI 61693	Decrease	25.2
Protein	RAC875_c8842_724	7D	37	3.8	PI 61693	Increase	22.4

**Table 5:** Expression of candidate genes in 14 DAF flag leaves located within 7D: 62,106,312–73,745,641 of IWSGC refseqv1.0 ^a^.

Gene	Protein Name (Uniprot)	Berkut	PI 61693
TraesCS7D02G103300	B box-type domain-containing protein	22.99	18.18
**TraesCS7D02G103500**	**2Fe-2S ferredoxin-type domain-containing protein**	**262.43**	**248.63**
TraesCS7D02G103800	Transcription elongation factor 1 homolog	18.11	64.31
TraesCS7D02G104400	Bacterial surface antigen (D15) domain-containing protein	17.77	13.59
TraesCS7D02G105500	Proteasome subunit beta	53.68	59.18
TraesCS7D02G105700	DUF676 domain-containing protein	9.35	12.57
TraesCS7D02G105900	Thiol methyltransferase 2	15.92	25.63
TraesCS7D02G106400	Mitogen-activated protein kinase	13.95	11.48
TraesCS7D02G106500	Uncharacterized protein	5.59	8.98
TraesCS7D02G107500	RING-type domain-containing protein	9.27	15.53
TraesCS7D02G107600	Uncharacterized protein	22.65	37.82
TraesCS7D02G108200	AAA+ ATPase domain-containing protein	8.18	6.70
TraesCS7D02G111300	Succinate dehydrogenase assembly factor 4, mitochondrial	11.44	18.07
TraesCS7D02G111500	Phytochromobilin:ferredoxin oxidoreductase, chloroplastic	4.42	11.04
**TraesCS7D02G111600**	**Putative kinase inhibitor WFT, Flowering locus T protein**	**50.58**	**219.66**
TraesCS7D02G111800	GDSL esterase/lipase	26.48	82.82
TraesCS7D02G113200	PIN domain-containing protein	9.38	10.93
TraesCS7D02G115400	Histone H3.2	6.02	5.92
**TraesCS7D02G117800**	**Starch synthase wSsI-1, chloroplastic/amyloplastic**	**42.82**	**28.37**
TraesCS7D02G118100	UBA domain-containing protein	9.87	13.88

^a^ 165 candidate genes were identified within 7D: 62,106,312–73,743,194. Candidate genes were only included within this table if they had expression values ≥ 5.0. Tissue was collected in the 2019 Original Starch Survey Field Trial. Samples consist of composites of three biological reps, where each biological rep was the bulk of three flag leaves collected from individual plants. Therefore, values are from single samples, but represent a composite of nine plants for each genotype. The putative protein function may be found in Table S3.

**Table 6:** Average temperatures (°C) for the 2019–2021 growing seasons.

	May	June	July	August
2019	25.4	21.5	25.9	27.0
2020	17.3	21.2	26.9	28.5
2021	15.6	26.6	31.2	26.2

## Data Availability

The data supporting the findings of this study are available from the corresponding author upon reasonable request.

## Supplementary Materials

The following supporting information can be downloaded at: https://www.mdpi.com/article/10.3390/plants14040512/s1, Table S1: Leaf and seed starch survey of modern wheat cultivars and landraces at 14 DAF. Table S2: Growth and yield parameters for RILs within a Berkut/PI 61693 population combined over two years. Table S3: Expression of candidate genes located within 7D: 62,106,312–73,745,641 using IWSGC refseqv1.0.

## Abbreviations

AGPase: ADP-glucose pyrophosphorylase; DAF, days after flowering; *FT1*, *Flowering Locus T1*; RIL, recombinant inbred line; QTL, quantitative trait loci.
