# Supplementary material for: Plant Productivity and Leaf Starch During Grain Fill Is Linked to QTL Containing Flowering Locus T1 (FT1) in Wheat (Triticum aestivum L.)
Source: Plants (Basel). 2025 Feb 7;14(4):512. doi: 10.3390/plants14040512 (PMC11858846; doi:10.3390/plants14040512)
Supplement: Supplementary file 1 [file plants-14-00512-s001.zip › plants-3410713-supplementary.pdf]

Table S1. Leaf and seed starch survey of modern wheat cultivars and landraces at 14 DAF.

ID	PI No.	NAM No.	Genetic Cluster	Leaf Starch (ug mg^{-1} DW)	Seed Starch (% DW)
Landraces					
**LDRC 22**	**PI 61693**	**-**	**2**	**20.34 ± 3.40**	**79.2 ± 2.2**
LDRC 15	PI 8813	16	1	11.52 ± 1.31	75.5 ± 2.5
LDRC 65	PI 283147	3	2	10.26 ± 1.38	75.7 ± 0.4
LDRC 10	Cltr 15144	18	4	7.51 ± 2.36	77.6 ± 3.5
LDRC 28	PI 94567	20	1	7.28 ± 1.30	68.7 ± 3.6
LDRC 48	PI 220431	29	1	5.32 ± 0.69	70.1 ± 3.4
LDRC 89	PI 470817	6	2	5.26 ± 1.92	72.9 ± 4.6
LDRC 47	PI 210945	28	1	4.84 ± 4.83	65.3 ± 0.1
LDRC 9	Cltr 15134	25	4	4.59 ± 1.28	81.3 ± 2.8
LDRC 31	PI 153785	-	1	4.27 ± 0.83	68.6 ± 0.7
LDRC 41	PI 192001	23	1	3.60 ± 0.36	74.0 ± 1.2
LDRC 33	PI 166333	-	1	3.01 ± 0.81	70.3 ± 3.7
LDRC 103	PI 572692	2	3	2.43 ± 0.72	71.9 ± 2.4
LDRC 25	PI 82469	17	1	2.23 ± 0.60	69.3 ± 4.0
LDRC 16	PI 9791	10	4	2.09 ± 0.67	86.3 ± 2.7
LDRC 42	PI 192147	24	4	1.97 ± 0.78	77.1 ± 1.0
LDRC 74	PI 366716	4	4	1.93 ± 1.12	67.4 ± 2.7
LDRC 2	Cltr 4175	11	4	1.86 ± 0.43	79.5 ± 1.6
LDRC 5	Cltr 11223	12	3	1.78 ± 0.15	65.7 ± 2.5
LDRC 23	PI 70613	14	3	1.72 ± 0.02	64.3 ± 3.5
LDRC 81	PI 382150	5	3	1.66 ± 0.09	75.2 ± 2.8
LDRC 53	PI 262611	13	4	1.66 ± 0.32	75.7 ± 1.9
LDRC 19	PI 43355	19	1	1.61 ± 0.64	74.7 ± 10.8
LDRC 99	PI 565213	7	3	1.55 ± 0.24	82.4 ± 1.4
LDRC 91	PI 477870	-	3	1.40 ± 0.49	74.3 ± 1.9
LDRC 43	PI 192569	27	2	1.39 ± 0.13	78.2 ± 0.8
LDRC 3	Cltr 7635	15	4	0.97 ± 0.23	73.1 ± 3.2
LDRC 37	PI 1585715	26	1	0.96 ± 0.37	71.6 ± 1.1
LDRC 60	PI 278297	30	1	0.81 ± 0.06	72.7 ± 2.4
LDRC average				3.89 ± 0.75	73.6 ± 1.0
Modern Cultivars	Release Year				
Marquis	1910			0.56 ± 0.05	76.0 ± 1.1
Ceres	1926			2.37 ± 0.20	82.6 ± 6.4
Thatcher	1934			1.85 ± 0.31	71.7 ± 0.9
Pilot	1939			3.17 ± 1.34	62.9 ± .3
Newthatch	1944			2.13 ± 0.53	74.4 ± 1.5
Rescue	1946			5.99 ± 0.82	72.9 ± .2
Chinook	1952			1.65 ± 0.26	79.5 ± 3.0
Centana	1958			1.70 ± 0.48	70.2 ± 1.1
Sawtana	1961			3.76 ± 2.38	71.2 ± 1.6

Fortuna	1966		3.82 ± 0.34	80.5 ± 1.5
Era	1970		2.25 ± 0.43	66.6 ± 2.1
Shortana	1971		2.42 ± 1.24	64.7 ± .4
Lew	1976		2.98 ± 0.97	64.3 ± 3.3
Newana	1976		1.02 ± 0.26	71.6 ± 2.0
Amidon	1988		2.69 ± 0.31	63.2 ± 2.6
HiLine	1991		2.26 ± 1.14	68.9 ± .2
McNeal	1994		0.83 ± 0.32	63.7 ± 2.7
Westbred 936	1998		3.39 ± 0.48	76.3 ± 1.2
Reeder	1999		2.15 ± 0.27	79.3 ± .8
**Berkut**	**2002**		**3.57 ± 0.49**	**72.8 ± 4.8**
Vida	2005		4.04 ± 0.98	72.8 ± 4.8
Modern cultivar average			2.60 ± 0.27	73.4 ± 2.5

Table S2. Growth and yield parameters for RILs within a Berkut/PI 61693 population combined over two years[a]

RIL#	Days to Anthesis	Leaf Starch (µg starch mg^{-1} DW)	Height (cm)	Biomass (g)	Seed Wt (g)	Harvest Index	ISW (mg)	Seed No	Seed Protein (%)	***FT-D1 Allele***
PI 61693	60.67	23.74	68.86	49.60	16.35	0.33	38.00	437.64	14.60	*FT-D1*
Berkut	64.50	5.72	69.30	58.91	18.99	0.32	37.15	513.45	14.18	*FT-D1ΔG*
**3**	61.67	10.08	62.69	47.07	14.17	0.30	31.25	466.01	13.10	*FT-D1ΔG*
**4**	62.00	9.77	74.37	66.36	20.51	0.31	38.10	536.28	13.97	*FT-D1ΔG*
**5**	62.83	11.40	66.23	68.16	21.49	0.32	27.68	795.59	13.08	*FT-D1ΔG*
**8**	61.17	7.19	66.76	81.09	26.64	0.33	32.92	803.97	14.43	*FT-D1ΔG*
**10**	63.67	15.55	74.14	73.31	23.87	0.32	34.96	674.07	13.93	*FT-D1ΔG*
**14**	63.50	11.70	63.89	63.81	21.62	0.33	28.12	745.10	12.80	*FT-D1ΔG*
**15**	64.33	9.22	70.39	68.30	22.99	0.33	37.10	624.11	13.83	*FT-D1ΔG*
**16**	66.00	7.19	84.12	125.86	40.02	0.32	39.87	998.69	13.50	*FT-D1ΔG*
**21**	62.17	11.14	72.60	71.85	23.42	0.33	33.97	702.18	13.48	*FT-D1ΔG*
**22**	64.00	12.17	67.46	64.09	19.31	0.30	36.03	538.51	14.28	*FT-D1ΔG*
**23**	62.17	11.82	79.75	81.53	26.30	0.32	35.28	740.14	13.00	*FT-D1ΔG*
**25**	65.83	6.99	76.97	83.69	25.67	0.31	39.16	646.79	13.35	*FT-D1ΔG*
**28**	63.33	6.95	73.54	74.75	24.47	0.33	34.24	710.30	13.68	*FT-D1ΔG*
**30**	62.17	8.76	64.29	75.60	25.87	0.34	35.64	730.86	13.85	*FT-D1ΔG*
**32**	62.33	8.47	85.74	92.12	28.19	0.31	40.65	688.99	14.58	*FT-D1ΔG*
**39**	62.33	12.45	71.86	75.38	24.23	0.32	35.68	663.86	12.98	*FT-D1ΔG*
**43**	60.83	10.49	69.53	43.24	13.93	0.32	34.07	411.78	13.15	*FT-D1ΔG*
**47**	63.67	8.97	75.11	80.45	25.74	0.32	35.22	672.45	12.40	*FT-D1ΔG*
**49**	63.50	7.54	72.24	75.22	24.22	0.32	36.74	662.39	12.73	*FT-D1ΔG*
**51**	63.50	7.27	85.72	72.10	21.35	0.30	40.20	526.03	14.93	*FT-D1ΔG*
**53**	60.67	8.90	61.75	52.31	17.18	0.33	35.65	489.41	13.60	*FT-D1ΔG*
**54**	62.00	11.47	73.67	65.66	23.33	0.35	34.81	667.05	12.93	*FT-D1ΔG*
**58**	60.17	12.59	74.40	73.00	23.43	0.32	35.20	681.48	14.15	*FT-D1ΔG*
**59**	61.33	14.10	74.51	72.45	23.02	0.32	33.01	695.71	13.63	*FT-D1ΔG*
**62**	65.50	6.90	78.94	93.50	29.22	0.31	37.80	769.15	15.30	*FT-D1ΔG*
**66**	62.00	14.75	69.40	65.68	18.45	0.28	41.94	435.46	14.70	*FT-D1ΔG*

**67**	60.17	10.07	65.49	44.64	14.10	0.32	33.87	422.65	13.53	*FT-D1ΔG*
**72**	61.67	5.50	70.54	69.96	21.28	0.30	36.28	591.99	14.60	*FT-D1ΔG*
**73**	62.67	6.24	82.89	70.19	21.51	0.30	36.21	580.82	14.00	*FT-D1ΔG*
**74**	63.50	8.88	77.41	76.15	23.18	0.31	41.19	556.81	13.70	*FT-D1ΔG*
**Ave**	62.69	9.82	72.88	72.25	22.96	0.32	35.76	640.96	13.70	*FT-D1ΔG*
**SE**	0.28	0.48	1.21	2.85	0.92	0.00	0.61	23.66	0.13	
**2**	61.67	19.39	81.42	68.93	22.77	0.33	36.00	628.28	13.25	*FT-D1*
**7**	61.00	10.39	75.37	56.40	17.35	0.31	36.41	487.41	14.83	*FT-D1*
**9**	61.67	14.30	61.42	58.59	18.48	0.32	28.68	645.93	14.53	*FT-D1*
**11**	62.67	16.01	70.83	57.18	18.17	0.32	36.53	494.65	15.18	*FT-D1*
**12**	65.83	8.45	64.58	68.37	22.59	0.33	34.05	665.08	13.70	*FT-D1*
**13**	63.50	13.35	57.68	46.74	14.20	0.30	35.82	412.28	15.20	*FT-D1*
**17**	64.67	11.70	77.31	81.27	26.57	0.32	35.49	743.72	14.43	*FT-D1*
**18**	61.67	10.51	59.48	54.73	17.43	0.32	32.20	535.40	12.98	*FT-D1*
**20**	61.17	14.95	51.94	25.37	8.10	0.32	33.62	242.93	13.48	*FT-D1*
**24**	61.17	10.28	71.08	65.45	21.65	0.33	36.32	577.12	14.68	*FT-D1*
**26**	62.17	14.12	55.38	47.64	15.88	0.33	36.58	440.67	13.00	*FT-D1*
**27**	61.50	6.56	79.21	60.94	18.61	0.31	37.70	493.44	15.00	*FT-D1*
**31**	61.50	15.31	59.99	37.22	11.08	0.30	33.53	349.57	15.93	*FT-D1*
**34**	61.50	18.64	87.86	65.74	19.22	0.29	40.01	491.40	16.33	*FT-D1*
**35**	60.83	13.42	58.75	38.85	12.24	0.32	31.17	353.15	14.43	*FT-D1*
**36**	61.17	9.59	65.12	60.07	19.73	0.33	34.78	567.65	13.48	*FT-D1*
**37**	61.33	12.80	60.95	44.63	11.14	0.35	35.03	307.22	15.00	*FT-D1*
**38**	61.67	12.63	71.74	53.35	15.65	0.29	42.80	367.10	15.40	*FT-D1*
**40**	60.83	9.23	62.76	55.61	18.18	0.33	37.28	489.84	15.40	*FT-D1*
**41**	61.33	20.40	69.90	28.35	6.94	0.24	34.10	201.63	15.23	*FT-D1*
**42**	62.50	17.55	69.33	59.23	17.44	0.29	39.60	445.26	15.98	*FT-D1*
**45**	64.17	18.48	72.43	47.24	13.59	0.29	39.91	339.07	16.20	*FT-D1*
**46**	63.00	11.06	76.94	54.70	17.15	0.31	36.20	476.42	14.98	*FT-D1*
**52**	60.50	17.61	58.21	51.33	16.61	0.32	37.26	462.44	14.20	*FT-D1*
**55**	60.17	11.62	74.47	63.05	20.17	0.32	41.84	495.46	13.85	*FT-D1*

**57**	61.00	8.98	70.13	63.54	21.92	0.34	39.46	571.27	13.38	*FT-D1*
**60**	63.00	11.65	76.08	72.57	23.02	0.31	40.91	555.11	14.23	*FT-D1*
**61**	63.00	12.79	79.44	69.33	21.98	0.32	46.40	480.43	12.28	*FT-D1*
**64**	60.50	13.88	56.48	38.89	12.68	0.33	36.47	358.49	13.63	*FT-D1*
**65**	62.50	10.47	71.30	52.85	15.76	0.30	40.39	395.09	16.68	*FT-D1*
**69**	61.00	11.36	62.49	42.81	12.81	0.30	37.57	373.89	17.05	*FT-D1*
**70**	60.67	9.72	61.92	47.01	15.42	0.33	34.60	444.88	13.63	*FT-D1*
**71**	60.50	6.44	65.59	38.10	12.60	0.33	41.26	305.49	13.73	*FT-D1*
**75**	59.67	11.25	65.59	31.99	8.90	0.28	32.59	271.51	15.40	*FT-D1*
**Ave**	61.79	12.79	67.74	53.18	16.65	0.31	36.84	454.98	14.61	*FT-D1*
**SE**	0.23	0.61	1.47	2.25	0.80	0.00	0.62	21.37	0.20	
**p-value**	0.01	0.000	0.01	0.000	0.000	0.40	0.22	0.000	0.000	
**56**	61.33	10.60	70.16	51.83	16.65	0.32	43.15	384.65	15.48	Segregating
**1**	62.33	8.68	65.99	67.73	21.33	0.31	36.07	588.95	13.63	Segregating
**6**	60.67	11.87	67.53	62.15	19.31	0.31	43.06	460.19	13.78	Segregating
**19**	63.17	10.08	69.45	84.62	27.09	0.32	36.26	756.27	14.43	Segregating
**33**	62.83	11.25	67.16	67.80	22.34	0.32	36.39	593.18	13.85	Segregating
**63**	60.83	8.29	56.27	59.03	19.13	0.32	33.88	571.14	15.03	Segregating
**68**	61.83	9.41	64.62	58.16	18.15	0.31	32.74	551.53	13.93	Segregating

[a] Values represent average ± SE with n = 2 for each genotype except days to anthesis and leaf starch where n = 3. Leaf starch was measured in flag leaves collected mid-afternoon at 14 DAF. Growth parameters such as biomass and seed weight are reported on an individual plant basis. ISW represents individual seed weight. Protein is based on 12% moisture.

Table S3. Candidate genes located within 7D:66345566-69266821 within IWSGC refseqv1.0a

Gene	7D Location (Ensembl)	Protein Name (Uniprot)	Putative Protein Function	Berkut	PI 61693
TraesCS7D02G102300	62,106,312 - 62,107,184	Very-long-chain 3-oxoacyl-CoA synthase	Fatty acid biosynthetic process	0.00	0.00
TraesCS7D02G102400	62,121,961 - 62,124,043	3-ketoacyl-CoA synthase	Fatty acid biosynthetic process	0.00	0.00
TraesCS7D02G102500	62,155,121 - 62,162,201	Saccharopine dehydrogenase NADP binding domain-containing protein	Glycolipid biosynthetic process	0.00	0.00
TraesCS7D02G102600	62,169,641 - 62,171,640	B-keto acyl reductase	Ketoreductase activity	0.00	0.00
TraesCS7D02G102700	62,190,909 - 62,197,160	Aldehyde oxygenase (deformylating)	Iron ion binding, lipid biosynthetic process, response to stress	0.00	0.03
TraesCS7D02G102800	62,259,247 - 62,260,596	3-ketoacyl-CoA synthase	Fatty acid biosynthetic process	0.00	0.00
TraesCS7D02G102900	62,280,083 - 62,281,525	3-ketoacyl-CoA synthase	Fatty acid biosynthetic process	0.00	0.00
TraesCS7D02G103000	62,929,112 - 62,930,429	Uncharacterized protein	Ketoreductase activity	0.00	0.00
TraesCS7D02G103100	62,940,310 - 62,942,721	Aldehyde oxygenase (deformylating)	Iron ion binding, lipid biosynthetic process, response to stress	0.00	0.00
TraesCS7D02G103200	63,303,222 - 63,306,821	Rhomboid-like protein	Membrane protein	2.62	5.73
TraesCS7D02G103300	63,325,536 - 63,331,403	B box-type domain-containing protein	Regulation of DNA-templated transcription	22.99	18.18
TraesCS7D02G103400	63,377,033 - 63,378,385	Serpin domain-containing protein	Serine-type endopeptidase inhibitor activity	0.00	0.00
**TraesCS7D02G103500**	63,381,222 - 63,382,771	**2Fe-2S ferredoxin-type domain-containing protein**	**P450-containing electron transport chain**	**262.43**	**248.63**
TraesCS7D02G103700	63,430,703 - 63,435,417	Ubiquinone biosynthesis O-methyltransferase COQ3, mitochondrial	Ubiquinone biosynthetic pathway.	3.48	5.02
TraesCS7D02G103800	63,435,738 - 63,437,937	Transcription elongation factor 1 homolog	Transcription elongation by RNA polymerase II	18.11	64.31
TraesCS7D02G103900	63,442,325 - 63,446,365	Pentacotripeptide-repeat region of PRORP domain-containing protein	RNA processing*	0.23	0.84
TraesCS7D02G104000	63,447,832 - 63,449,659	GDSL esterase/lipase	Hydrolase activity, acting on ester bonds	0.06	0.00
TraesCS7D02G104100	63,453,206 - 63,455,816	GDSL esterase/lipase	Hydrolase activity, acting on ester bonds	0.00	0.00
TraesCS7D02G104200	63,462,131 - 63,464,888	F-box domain-containing protein	Protein-protein interactions*	0.17	0.11
TraesCS7D02G104300	63,468,787 - 63,470,350	GDSL esterase/lipase	Hydrolase activity, acting on ester bonds	0.00	0.00
TraesCS7D02G104400	63,483,494 - 63,485,663	Bacterial surface antigen (D15) domain-containing protein	--	17.77	13.59
TraesCS7D02G104500	63,555,076 - 63,564,924	WAT1-related protein	Transmembrane transporter activity	0.00	0.00
TraesCS7D02G104600	63,600,323 - 63,604,539	WAT1-related protein	Transmembrane transporter activity	0.00	0.00
TraesCS7D02G104700	63,605,064 - 63,610,325	WAT1-related protein	Transmembrane transporter activity	0.00	0.00
TraesCS7D02G104800	63,611,408 - 63,611,611			0.00	0.00
TraesCS7D02G104900	63,639,888 - 63,644,990	WAT1-related protein	Transmembrane transporter activity	0.00	0.00
TraesCS7D02G105000	63,648,050 - 63,648,253	Subtilisin inhibitor 1	Serine-type endopeptidase inhibitor activity, response to wounding	0.00	0.00
TraesCS7D02G105100	63,730,790 - 63,737,106	Sugar phosphate transporter domain-containing protein	UDP-glucose transmembrane transport	1.44	2.64
TraesCS7D02G105200	63,921,794 - 63,923,164	DUF1618 domain-containing protein	--	0.00	0.00
TraesCS7D02G105300	63,929,748 - 63,933,652	Uncharacterized protein	--	0.09	0.61
TraesCS7D02G105500	63,936,271 - 63,939,170	Proteasome subunit beta	Proteasomal protein catabolic process	53.68	59.18
TraesCS7D02G105600	63,940,572 - 63,943,730	AAA+ ATPase domain-containing protein	ADP binding, defense response, response to other organism	1.09	1.39
TraesCS7D02G105700	63,944,946 - 63,948,997	DUF676 domain-containing protein	Cellular lipid metabolic process	9.35	12.57
TraesCS7D02G105800	63,995,790 - 63,996,338	Zinc finger GRF-type domain-containing protein	DNA-binding*	0.00	0.00
TraesCS7D02G105900	64,007,540 - 64,011,402	Thiol methyltransferase 2	Methylation	15.92	25.63
TraesCS7D02G106000	64,011,871 - 64,013,981	MAX2	Ubiquination, SCF ubiquitin ligase complex	1.73	1.47
TraesCS7D02G106100	64,244,283 - 64,249,047	AAA+ ATPase domain-containing protein	ADP binding, defence response to another organism	0.34	0.08
TraesCS7D02G106200	64,265,813 - 64,266,996	Rx N-terminal domain-containing protein	ADP binding, defence response to another organism	2.72	1.13

TraesCS7D02G106300	64,267,131 - 64,269,076	NB-ARC domain-containing protein	ADP binding, defence response to another organism	2.74	4.11
TraesCS7D02G106400	64,270,551 - 64,277,804	Mitogen-activated protein kinase	Protein serine/threonine kinase activity, intracellular signal transduction	13.95	11.48
TraesCS7D02G106500	64,379,888 - 64,385,076	Uncharacterized protein	--	5.59	8.98
TraesCS7D02G106600	64,436,698 - 64,446,546	MIKC-type MADS-box transcription factor	Regulation of transcription by RNA polymerase II	0.00	0.00
TraesCS7D02G106800	64,466,713 - 64,468,253	F-box domain-containing protein	TRANSMEMBRANE PROTEIN 1 hit	0.05	0.09
TraesCS7D02G106900	64,471,702 - 64,488,450	MIKC-type MADS-box transcription factor	Regulation of transcription by RNA polymerase II	0.00	0.00
TraesCS7D02G107000	64,496,924 - 64,497,294	MADS-box domain-containing protein	Regulation of transcription by RNA polymerase II	0.00	0.00
TraesCS7D02G107100	64,514,788 - 64,515,697	K-box domain-containing protein	Regulation of transcription by RNA polymerase II	0.00	0.00
TraesCS7D02G107200	64,527,342 - 64,528,011	MADS-box domain-containing protein	Regulation of transcription by RNA polymerase II	0.00	0.00
TraesCS7D02G107300	64,666,735 - 64,668,718	SIAH-type domain-containing protein	E3 ubiquitin-protein ligase that mediates ubiquitination and subsequent proteasomal degradation of target proteins.	0.08	0.00
TraesCS7D02G107400	64,682,005 - 64,682,496	AP2/ERF domain-containing protein	DNA-binding transcription factor activity	0.00	0.00
TraesCS7D02G107500	64,683,828 - 64,688,450	RING-type domain-containing protein	--	9.27	15.53
TraesCS7D02G107600	64,695,975 - 64,699,486	Uncharacterized protein	Calmodulin binding	22.65	37.82
TraesCS7D02G107700	64,730,992 - 64,734,629	Post-GPI attachment to proteins factor 3	Involved in the lipid remodeling steps of GPI-anchor maturation	3.62	4.11
TraesCS7D02G107800	64,735,107 - 64,736,591	DUF295 domain-containing protein	--	0.03	0.12
TraesCS7D02G107900	64,737,046 - 64,740,662	EIG-I24	Alkaloid biosynthesis; taxol biosynthesis	2.08	5.74
TraesCS7D02G108000	64,808,166 - 64,809,600	Uncharacterized protein	Transcription regulation	0.00	0.00
TraesCS7D02G108100	64,829,010 - 64,830,600	Uncharacterized protein	Transcription regulation	0.00	0.00
TraesCS7D02G108200	64,885,232 - 64,891,403	AAA+ ATPase domain-containing protein	ATP binding, ATP hydrolysis activity	8.18	6.70
TraesCS7D02G108300	64,893,828 - 64,894,121	Uncharacterized protein	Receptor serine/threonine kinase binding, cell-cell signaling involved in cell fate commitment	0.00	0.00
TraesCS7D02G108400	64,912,484 - 64,912,771	Uncharacterized protein	Receptor serine/threonine kinase binding, cell-cell signaling involved in cell fate commitment	0.00	0.00
TraesCS7D02G108500	65,390,393 - 65,390,769	Uncharacterized protein	Receptor serine/threonine kinase binding, cell-cell signaling involved in cell fate commitment	0.00	0.00
TraesCS7D02G108600	65,399,270 - 65,399,986	SGNH hydrolase-type esterase domain-containing protein	Hydrolysis of ester bonds*	0.00	0.00
TraesCS7D02G108700	65,437,262 - 65,438,587	Uncharacterized protein	Oxidoreductase	0.00	0.00
TraesCS7D02G108800	65,447,910 - 65,448,806	Chitinase	Chitin degredation*	0.00	0.00
TraesCS7D02G108900	65,599,420 - 65,601,414	Esterase	Hydrolysis of ester bonds*	1.02	0.42
TraesCS7D02G109000	65,626,594 - 65,626,842	Uncharacterized protein	Oxidoreductase	0.00	0.00
TraesCS7D02G109100	65,731,169 - 65,732,308	GDSL esterase/lipase	Hydrolase activity, acting on ester bonds	0.00	0.00
TraesCS7D02G109200	65,750,012 - 65,752,116	GDSL esterase/lipase	Hydrolase activity, acting on ester bonds	0.00	0.10
TraesCS7D02G109300	65,761,945 - 65,762,256	Transcription elongation factor 1 homolog	Transcription elongation factor implicated in the maintenance of proper chromatin structure in actively transcribed regions.	0.00	0.00
TraesCS7D02G109400	65,902,172 - 65,903,121	DUF569 domain-containing protein	Actin-crosslinking	0.00	0.00
TraesCS7D02G109500	65,937,306 - 65,940,311	DUF569 domain-containing protein	Actin-crosslinking	0.07	0.00
TraesCS7D02G109600	66,345,566 - 66,348,319	DUF569 domain-containing protein	Actin-crosslinking	0.00	0.00
TraesCS7D02G109700	66,373,919 - 66,376,031	Actin cross-linking protein, putative (DUF569)	Actin-crosslinking	0.00	0.00
TraesCS7D02G109800	66,399,827 - 66,400,583	F-box protein family-like	SCF-dependent proteasomal ubiquitin-dependent protein catabolic process	0.00	0.00
TraesCS7D02G109900	66,412,919 - 66,416,543	Actin cross-linking protein, putative (DUF569)	Actin-crosslinking	0.00	0.00
TraesCS7D02G110000	66,484,785 - 66,485,114	Transcription elongation factor 1-like protein	Transcription elongation by RNA polymerase II	0.00	0.00
TraesCS7D02G110100	66,513,516 - 66,513,830	Transcription elongation factor 1-like protein	Transcription elongation by RNA polymerase II	0.00	0.00

TraesCS7D02G110200	66,520,630 - 66,521,667	Werner Syndrome-like exonuclease	Nucleic acid binding	0.00	0.00
TraesCS7D02G110300	66,546,054 - 66,548,142	Actin cross-linking protein, putative (DUF569)	Actin-crosslinking	0.00	0.00
TraesCS7D02G110400	66,595,264 - 66,601,382	Sulfotransferase	Heparan sulfate proteoglycan biosynthetic process, enzymatic modification	0.48	7.48
TraesCS7D02G110500	66,690,292 - 66,694,641	Nuclear nucleic acid-binding protein C1D	Regulation of gene expression	1.73	2.09
TraesCS7D02G110600	66,695,801 - 66,699,035	WRKY transcription factor, putative	DNA-binding transcription factor activity	0.18	0.77
TraesCS7D02G110700	66,700,645 - 66,709,049	Long-Chain Acyl-CoA Synthetase	Fatty acid biosynthesis	1.12	2.90
TraesCS7D02G110800	66,817,979 - 66,818,437	Non-specific lipid-transfer protein	Lipid transport	0.12	0.00
TraesCS7D02G110879	66,821,216 - 66,821,641	Bifunctional inhibitor/plant lipid transfer protein/seed storage helical domain-containing protein	Lipid transport	0.00	0.00
TraesCS7D02G110900	66,822,443 - 66,831,771	Protoheme IX farnesyltransferase	Heme A biosynthetic process	3.77	4.86
TraesCS7D02G111000	66,833,144 - 66,833,605	Non-specific lipid-transfer protein	Lipid transport*	0.00	0.00
TraesCS7D02G111100	66,871,451 - 66,873,203	Hydroxysteroid dehydrogenase 3	Ketoreductase activity	0.00	0.00
TraesCS7D02G111200	66,953,256 - 66,958,978	O-acyltransferase WSD1	Triglyceride biosynthetic process	0.00	0.00
TraesCS7D02G111226	66,961,967 - 66,962,395	Uncharacterized transmembrane protein	--	0.00	0.00
TraesCS7D02G111300	66,978,198 - 66,979,979	Succinate dehydrogenase assembly factor 4, mitochondrial	Mitochondrial respiratory chain complex II assembly	11.44	18.07
TraesCS7D02G111400	66,987,743 - 66,989,152	ATP synthase subunit beta	ATP metabolism*	0.29	0.19
TraesCS7D02G111500	67,549,372 - 67,552,886	Phytochromobilin:ferredoxin oxidoreductase, chloroplastic	Porphyrin and chlorophyll metabolism*	4.42	11.04
**TraesCS7D02G111600**	**67,549,372 - 67,552,886**	**Putative kinase inhibitor WFT, Flowering locus T protein**	**Vegetative to reproductive phase transition of meristem**	**50.58**	**219.66**
TraesCS7D02G111700	68,517,948 - 68,518,403	VQ motif family protein	Regulation of plant development and stress responses*	0.00	0.25
TraesCS7D02G111800	68,790,196 - 68,792,445	GDSL esterase/lipase	Hydrolase activity, acting on ester bonds	26.48	82.82
TraesCS7D02G111900	68,793,538 - 68,799,889	Hexosyltransferase	Galactosyltransferase activity	0.86	2.75
TraesCS7D02G112000	68,801,675 - 68,803,318	Actin cross-linking protein, putative (DUF569)	Actin-crosslinking	0.00	0.14
TraesCS7D02G112100	68,805,651 - 68,805,980	Transcription elongation factor 1 homolog	Transcription elongation by RNA polymerase II	0.00	0.00
TraesCS7D02G112200	68,897,599 - 68,903,825	Actin cross-linking protein, putative (DUF569)	Actin-crosslinking	0.00	0.00
TraesCS7D02G112300	68,913,733 - 68,914,032	Transcription elongation factor 1 homolog	Transcription elongation by RNA polymerase II	0.00	0.00
TraesCS7D02G112400	68,923,883 - 68,924,140	Transcription elongation factor 1 homolog	Transcription elongation by RNA polymerase II	0.00	0.00
TraesCS7D02G112500	68,928,581 - 68,930,600	Protein of unknown function DUF569 domain containing protein	--	0.00	0.00
TraesCS7D02G112600	68,934,813 - 68,935,142	Transcription elongation factor 1 homolog	Transcription elongation by RNA polymerase II	0.00	0.00
TraesCS7D02G112700	68,983,488 - 68,985,494	Actin cross-linking protein, putative (DUF569)	Actin-crosslinking	0.00	0.00
TraesCS7D02G112800	69,041,586 - 69,044,020	Actin cross-linking protein, putative (DUF569)	Actin-crosslinking	0.00	0.00
TraesCS7D02G112900	69,041,586 - 69,044,020	Actin cross-linking protein, putative (DUF569)	Actin-crosslinking	0.00	0.00
TraesCS7D02G113000	69,200,980 - 69,201,502	Cyclin delta-3 G	Cell cycle regulation*	0.00	0.14
TraesCS7D02G113100	69,265,245 - 69,266,821	Basic helix loop helix (BHLH) family transcription factor	Regulation of DNA-templated transcription	0.03	0.11
TraesCS7D02G113200	69,590,037 - 69,593,428	PIN domain-containing protein	rRNA processing	9.38	10.93
TraesCS7D02G113300	69,593,549 - 69,596,887	Glyoxalase/fosfomycin resistance/dioxygenase domain-containing		1.51	1.58
TraesCS7D02G113400	69,642,872 - 69,644,228	Legume lectin domain-containing protein	Membrane protein	0.00	0.00
TraesCS7D02G113500	69,661,893 - 69,663,516	DUF295 domain-containing protein		0.04	0.16
TraesCS7D02G113600	69,774,165 - 69,779,079	Subtilisin-like protease	Serine protease	0.00	0.06
TraesCS7D02G113700	69,781,253 - 69,783,939	Subtilisin-like protease	Serine-type endopeptidase activity	0.00	0.00
TraesCS7D02G113800	69,798,911 - 69,802,797	Subtilisin-like protease	Serine-type endopeptidase activity	0.00	0.23

TraesCS7D02G113900	69,804,752 - 69,808,107	Subtilisin-like protease	Serine-type endopeptidase activity	0.00	0.00
TraesCS7D02G114000	69,810,359 - 69,813,142	Subtilisin-like protease	Serine protease	0.07	0.13
TraesCS7D02G114100	69,815,233 - 69,823,208	ABC transporter C family member 10	ATP hydrolysis activity	2.10	4.84
TraesCS7D02G114200	69,867,607 - 69,875,984	ABC transporter C family member 10	ATP hydrolysis activity, transmembrane transport	1.39	3.75
TraesCS7D02G114300	69,936,681 - 69,942,549	Uncharacterized protein	ABC Transporter activity	0.11	0.04
TraesCS7D02G114400	69,954,266 - 69,955,747	DUF295 domain-containing protein	--	3.16	1.92
TraesCS7D02G114500	70,048,437 - 70,049,669	DUF295 domain-containing protein	--	1.52	2.02
TraesCS7D02G114600	70,069,947 - 70,074,729	Uncharacterized protein	--	0.66	1.04
TraesCS7D02G114700	70,130,695 - 70,131,162	Uncharacterized protein	--	1.09	0.79
TraesCS7D02G114800	70,151,658 - 70,154,174	DUF295 domain-containing protein	--	2.20	2.60
TraesCS7D02G114900	70,679,243 - 70,679,784	RING-type E3 ubiquitin transferase	Mediates the transfer of ubiquitin from an E2 enzyme to a substrate protein	0.00	0.00
TraesCS7D02G115000	70,735,509 - 70,736,755	DUF295 domain-containing protein	--	1.88	1.18
TraesCS7D02G115100	70,745,497 - 70,754,564	ABC transporter C family member 10	ATP hydrolysis activity, transmembrane transport	0.19	0.47
TraesCS7D02G115200	70,820,277 - 70,821,652	Uncharacterized protein	Chloroplast, acyl-[acyl-carrier-protein] hydrolase activity	0.00	0.00
TraesCS7D02G115300	70,826,255 - 70,833,932	ABC transporter C family member 10	ATP hydrolysis activity, transmembrane transport	0.51	0.22
TraesCS7D02G115400	70,904,738 - 70,905,401	Histone H3.2	Core component of nucleosome	6.02	5.92
TraesCS7D02G115500	71,053,327 - 71,055,332	Dirigent protein	Biosynthesis of lignans, flavonolignans, and alkaloids	0.00	0.00
TraesCS7D02G115600	71,082,540 - 71,084,443	Dirigent protein	Biosynthesis of lignans, flavonolignans, and alkaloids	0.00	0.00
TraesCS7D02G115700	71,091,110 - 71,093,081	Dirigent protein	Biosynthesis of lignans, flavonolignans, and alkaloids	0.00	0.00
TraesCS7D02G115800	71,106,511 - 71,108,436	Dirigent protein	Biosynthesis of lignans, flavonolignans, and alkaloids	0.00	0.00
TraesCS7D02G115900	71,211,224 - 71,217,924	ABC transporter C family member 10	ATP hydrolysis activity, transmembrane transport	0.02	0.16
TraesCS7D02G116000	71,317,300 - 71,320,652	Uncharacterized protein	ATP hydrolysis activity, transmembrane transport	0.12	0.31
TraesCS7D02G116100	71,342,666 - 71,350,177	ABC transporter C family member 10	ATP hydrolysis activity, transmembrane transport	0.15	0.28
TraesCS7D02G116200	71,353,691 - 71,363,523	ABC transporter C family member 10	ATP hydrolysis activity, transmembrane transport	0.05	0.00
TraesCS7D02G116300	71,543,576 - 71,552,110	ABC transporter C family member 10	ATP hydrolysis activity, transmembrane transport	2.17	3.32
TraesCS7D02G116500	71,628,511 - 71,632,268	AAA+ ATPase domain-containing protein	ADP binding, defence response to another organism	0.00	0.00
TraesCS7D02G116600	71,812,505 - 71,813,150	Histone H3.2	Core component of nucleosome	1.66	2.34
TraesCS7D02G116700	71,837,050 - 71,839,162	Glycosyltransferase	Catalyze the synthesis of glycans and glycoconjugates	0.00	0.12
TraesCS7D02G116800	71,863,790 - 71,864,575	Histone H3.2	Core component of nucleosome	0.42	1.10
TraesCS7D02G116900	72,128,417 - 72,129,200	Histone H3.2	Core component of nucleosome	0.69	0.83
TraesCS7D02G117000	72,170,415 - 72,174,308	Uncharacterized Protein	ABC Transporter	0.16	0.63
TraesCS7D02G117100	72,216,363 - 72,216,773	Histone H3.2	Core component of nucleosome	0.42	1.10
TraesCS7D02G117200	72,261,884 - 72,270,700	Cytochrome P450	Oxidoreductase activity, Hormone metabolism	0.00	0.31
TraesCS7D02G117300	72,386,311 - 72,387,980	Cytochrome P450	Oxidoreductase activity, Hormone metabolism	0.00	0.00
TraesCS7D02G117400	72,434,671 - 72,436,471	Pectinesterase	Glycan metabolism, cell wall modification	0.00	0.00
TraesCS7D02G117500	72,478,840 - 72,479,663	Malectin-like domain-containing protein		0.00	0.00
TraesCS7D02G117600	72,522,125 - 72,523,125	AP2/ERF domain-containing protein	DNA-binding transcription factor activity	0.00	0.16
TraesCS7D02G117700	72,785,778 - 72,786,144	Uncharacterized protein	Microtubule binding	0.00	0.00
**TraesCS7D02G117800**	**72,936,703 - 72,947,152**	**Starch synthase wSsI-1, chloroplastic/amyloplastic**	**Glycan biosynthesis; starch biosynthesis**	**42.82**	28.37
TraesCS7D02G117900	72,950,111 - 72,951,971	Uncharacterized protein	Membrane protein	0.00	0.00
TraesCS7D02G118000	72,965,952 - 72,967,198	AP2/ERF domain-containing protein	DNA-binding transcription factor activity	0.00	0.00
TraesCS7D02G118100	73,107,102 - 73,114,816	UBA domain-containing protein	Ubiquitin-dependent protein catabolic process	9.87	13.88
TraesCS7D02G118200	73,116,721 - 73,117,131	Histone H3	Core component of nucleosome	3.04	5.64
TraesCS7D02G118300	73,128,509 - 73,130,805	(DL)-glycerol-3-phosphatase 2	Glycerol biosynthetic process	3.47	6.44
TraesCS7D02G118400	73,213,009 - 73,216,208	Actin cross-linking protein, putative (DUF569)	Mediate interactions between actin filaments	0.00	0.00
TraesCS7D02G118500	73,218,106 - 73,220,394	Actin cross-linking protein, putative (DUF569)	Mediate interactions between actin filaments	0.00	0.13

TraesCS7D02G118600	73,363,339 - 73,364,785	Uncharacterized protein	DNA binding	0.00	0.00
TraesCS7D02G118700	73,562,661 - 73,565,391	Actin cross-linking protein, putative (DUF569)	Mediate interactions between actin filaments	0.00	0.00
TraesCS7D02G118800	73,610,327 - 73,612,818	Actin cross-linking protein, putative (DUF569)	Mediate interactions between actin filaments	0.26	0.83
TraesCS7D02G118900	73,743,194 - 73,745,641	Actin cross-linking protein, putative (DUF569)	Mediate interactions between actin filaments	0.00	0.00
